# Protective Efficacy in Sheep of Adenovirus-Vectored Vaccines against Bluetongue Virus Is Associated with Specific T Cell Responses

**DOI:** 10.1371/journal.pone.0143273

**Published:** 2015-11-30

**Authors:** Verónica Martín, Elena Pascual, Miguel Avia, Lourdes Peña, Félix Valcárcel, Noemí Sevilla

**Affiliations:** Centro de Investigación en Sanidad Animal (CISA-INIA), Instituto Nacional de Investigación Agraria y Alimentaria, Valdeolmos, Madrid, Spain; University College Cork, IRELAND

## Abstract

Bluetongue virus (BTV) is an economically important *Orbivirus* of the *Reoviridae* family that causes a hemorrhagic disease in ruminants. Its control has been achieved by inactivated-vaccines that have proven to protect against homologous BTV challenge although unable to induce long-term immunity. Therefore, a more efficient control strategy needs to be developed. Recombinant adenovirus vectors are lead vaccine candidates for protection of several diseases, mainly because of their potency to induce potent T cell immunity. Here we report the induction of humoral and T-cell mediated responses able to protect animals against BTV challenge by recombinant replication-defective human adenovirus serotype 5 (Ad5) expressing either VP7, VP2 or NS3 BTV proteins. First we used the IFNAR^(-/-)^ mouse model system to establish a proof of principle, and afterwards we assayed the protective efficacy in sheep, the natural host of BTV. Mice were completely protected against BTV challenge, developing humoral and BTV-specific CD8^+^- and CD4^+^-T cell responses by vaccination with the different rAd5. Sheep vaccinated with Ad5-BTV-VP2 and Ad5-BTV-VP7 or only with Ad5-BTV-VP7 and challenged with BTV showed mild disease symptoms and reduced viremia. This partial protection was achieved in the absence of neutralizing antibodies but strong BTV-specific CD8^+^ T cell responses in those sheep vaccinated with Ad5-BTV-VP7. These data indicate that rAd5 is a suitable vaccine vector to induce T cell immunity during BTV vaccination and provide new data regarding the relevance of T cell responses in protection during BTV infection.

## Introduction

Bluetongue virus (BTV) is the prototype member of the genus *Orbivirus* within the *Reoviridae* family, transmitted to the vertebrate host by biting midges [1]. The genome is composed of ten segments of doubled-stranded RNA, encoding 7 structural- and 4 non-structural (NS) proteins that is enclosed by a complex capsid structure [2, 3]. The inner layer is constituted of VP3 (subcore) and VP7 (core), highly conserved proteins that play an important role in the structural integrity of the virus [4]. The outer capsid layer is composed of two major structural proteins, VP2 and VP5 [2, 5, 6]. VP2 is responsible for eliciting serotype-specific neutralizing antibodies [7], which have shown no cross-reactivity among the 26 different BTV serotypes circulating worldwide [8].

BTV infection in sheep results in acute disease associated with high morbidity and mortality, depending on the strain virulence and the sheep breed [9]. In cattle, goats and wild ruminants BTV infection is in most cases asymptomatic although they develop a prolonged viremia, representing a possible reservoir for BTV dissemination. Animals which recover from disease develop a long-lasting immunity, both of neutralizing antibodies [10] and cytotoxic T lymphocytes (CTL) [11]. Actually, both components of the immune response play a crucial role in protection against BTV, although cellular immunity seems to be decisive as BTV protection can be achieved in the absence of neutralizing antibodies [12, 13]. Interestingly, BTV infection and vaccination induces CTL in sheep able to cross-react with different BTV serotypes [14–17].

For controlling BTV infection, vaccination with live-attenuated vaccines has proven to be effective, eliciting a strong neutralizing antibody and cell-mediated immunity against homologous BTV infection [18]. However, several concerns have been raised against this vaccine strategy such as teratogenic effects, possibility of reassortment with wild-type viruses, and possible transmission to unvaccinated animals [19, 20]. Therefore, live-attenuated vaccines were replaced by inactivated-vaccines that have been proven to protect against homologous BTV challenge although inducing only short-term immunity [21, 22]. In addition, neither of these vaccines allow for discrimination between infected and vaccinated animals (DIVA). To overcome these problems, new strategies based on recombinant viral vector vaccines expressing BTV proteins have been developed [23–26]. In general, most of these vaccines express VP2 and are able to elicit a neutralizing antibody response but not a significant T-cell mediated BTV immune response. Among BTV proteins, VP7 is a major BTV group reactive antigen that contains CD8^+^ and CD4^+^ T cell epitopes [27, 28] that are conserved among different serotypes. Vaccination with recombinant capripox virus encoding VP7 [27] and recombinant canine adenovirus type 2 expressing VP7 [29] showed clinical protection against heterologous challenge, although the virus still replicated. In general, BTV NS proteins have mostly been associated with cellular immune responses [15, 16, 30]. Lobato et al. [31] showed that protection was improved when several recombinant BTV proteins were associated in the vaccine formulation. Therefore, this might indicate to include in the vaccine formulation an NS protein highly conserved between different serotypes might increase the rate of vaccine success.

In the current work, we have generated replication-defective human adenovirus 5 expressing VP7, VP2 or NS3 BTV proteins as a vaccination strategy for inducing strong immune responses, including cell-mediated immunity against BTV. VP2 and VP7 proteins were chosen based on containing the major neutralizing determinants of BTV [32] and T cell epitopes [28], respectively. NS3 protein was selected because antibodies against this protein are detected for longer than 200 dpi, which is much longer than the infectious period [33]. Therefore, to include NS3 might allow to evaluate whether this longer prevalence of antibodies against NS3 provide a more efficient vaccine. Initially, we used adult IFNAR(-/-) mice, that are a valid surrogate model for BTV vaccination studies [34], to evaluate whether the magnitude or quality of the immune response might be substantially affected (stimulatory or suppressive effect) by the expression of different BTV proteins and different combinations of these adenoviruses. Once the efficacy of our adenoviruses was determined, we tested in sheep, natural host of BTV infection, the efficiency of vaccination with the recombinant adenoviruses Our results demonstrated that vaccination with Ad5-BTV-VP7 resulted in significant CD4^+^ and CD8^+^ T cells response against BTV that confer partial protection in the absence of significant neutralizing antibodies.

## Material and Methods

### Ethical statement

All the animal experiments were carried out in a disease-secure isolation facility (BSL3) at the *Centro de Investigación en Sanidad Animal* (CISA), in strict accordance with the recommendations in the guidelines of the Code for Methods and Welfare Considerations in Behavioural Research with Animals (Directive 86/609EC; RD1201/2005) and all efforts were made to minimize suffering. Experiments were approved by the Committee on the Ethics of Animal Experiments (CEEA) (Permit number: 10/142792.9/12) of the Spanish *Instituto Nacional de Investigación y Tecnología Agraría y Alimentar*ia (INIA) and the “*Comisión de ética estatal de bienestar animal*” (Permit numbers: CBS2012/06 and PROEX 228/14).

### Cells and viruses

293 [HEK-293] cells (ATCC CRL-1573), Vero cells (ATCC CCL-81) and Baby Hamster Kidney (BHK, ATCC CRL-6281) cells were grown in Dulbecco’ modified Eagle’s medium (DMEM), supplemented with 10% Foetal Bovine Serum (FBS). Sheep thymus (ST) cells, an established cell line produced in our laboratory from primary thymus cells, were grown in DMEM supplemented with 10% FBS. BTV serotype 8 (Belgium/06) was used in all the experiments. BTV stocks were prepared as described previously [28]. Briefly, BHK cells were infected with BTV at multiplicity of infection (moi) of 0.1 and supernatants were collected at 48 hours post-infection (hpi). After 3 freeze/thaw cycles and 6 min sonication steps, the supernatants were clarified and stored at -80°C until use.

### Production of the recombinant adenoviruses Ad5-BTV-NS3, Ad5-BTV-VP2, Ad5-BTV-VP7 and Ad5-DsRed

The open reading frames encoding BTV-8 proteins NS3, VP2 and VP7 were amplified by RT-PCR and cloned into the pSIREN-EF1α plasmid and the derived plasmids pAd5-BTV-NS3, pAd5-BTV-VP2 and pAd5-BTV-VP7 were obtained with the technique described in [35]. Briefly, pSIREN-EF1α has been designed as a Donor Vector derived from the pSIREN-DNR-DsRed-Express (Clontech) to transfer the gene of interest and/or the red fluorescent protein (DsRed) to the adenoviral Acceptor Vector pLP-Adeno-X-PRLS (Clontech) by Cre-loxP mediated recombination. The DsRed-Express gene is positioned downstream of the immediate early promoter of cytomegalovirus. As a result of Cre mediated recombination, adenoviral plasmids are generated containing the gene of interest under the EF1α promoter, the DsRed protein under CMV promoter and the chloramphenicol resistance gene (CmR). The restriction enzyme PacI acting in these adenoviral plasmids releases the ITR allowing the generation of replication defective adenovirus, Ad5-BTV-NS3, Ad5-BTV-VP2, Ad5-BTV-VP7 and Ad5-DsRed, by transfecting [36] HEK 293A cells that provide in *trans* the E1 adenoviral replication and packaging functions. The recombinant viruses were amplified, purified and titrated using standard protocols and commercial kits (Clontech).

### RNA extraction and RT-PCR

BTV-8 infected cells were harvested at 48 hpi. These cells were frozen and thawed three times and sonicated at 20 cycles min^-1^ at 5w for 2 min and centrifuged at 1000 rpm for 5 min. Clarified viral stocks obtained as before were used for extracting total RNA using Trizol Reagent Solution (Invitrogen) following the manufacturer’s protocol. Reverse transcription of the NS3-, VP2- and VP7- mRNAs fragments was performed by two-step RT-PCR using SuperScript III reverse transcriptase (Invitrogen) and EHF DNApolymerase (Roche). To detect mRNA from recombinant adenoviruses HEK293A adenovirus-infected cells were harvested at 48 hpi and were used for extracting total RNA using Trizol (Invitrogen) following the manufacturer's protocol. Reverse transcription of the NS3-, VP2- and VP7- mRNAs fragments were performed. The complete NS3 and VP7 and partial VP2 mRNA (from nucleotide 550 to nucleotide 1592) were amplified. The sequence of primers employed in this work is available upon request.

### Western Blot analysis

HEK293A cells were seeded in M-24 well plates and they were infected with Ad5-NS3, Ad5-VP2 and Ad5-DsRed at a moi of 1. Twenty four hours post-infection the cells were lysed and harvested in Laemmli buffer and analysed by Western blot with a polyclonal serum from BTV-8 infected mice or sheep. Briefly, equivalent amounts of whole cell extracts were electrophoresed on SDS-10% polyacrylamide gels and transferred to nitrocellulose membranes. The membranes were incubated with a 1:150 dilution of the polyclonal sera, respectively and then with a 1:5,000 dilution of peroxidase-labeled anti-mouse serum (GE Life Sciences), and the proteins were detected with the ECL system (GE Life Sciences) according to the manufacturer's recommendations.

### Immunofluorescence microscopy

Semi-confluent Vero cells and ST cell monolayers were infected with Ad5-BTV-NS3, Ad5-BTV-VP2, Ad5-BTV-VP7 and Ad5-DsRed (moi of 10). At 48 hpi, the monolayers were fixed using methanol–acetone or 4% paraformaldehyde and proteins detected by indirect immunofluorescence using anti-BTV-8 polyclonal antibodies generated in our laboratory, followed by rabbit anti-sheep Alexa-488 secondary antibody (Bionova). Nuclei were counterstained using DAPI (Invitrogen). Samples were observed under fluorescence microscope (Olympus CKX41).

### Mice vaccination

Female (7–8 week-old) IFN α/βR^o/o^ IFNAR^(-/-)^ mice [37] on a C57BL/6 genetic background were housing in groups of 5 mice/cage of 834 cm2 of floor area and 19 cm high in the Animal Facilities of CISA-INIA. Bedding is provided with a minimum of 2 cm depth. Mice were immunized intramuscularly twice (at two weeks interval) with 10^8^ infectious units (IU) of Ad5-BTV-VP2 + Ad5-BTV-VP7 + Ad5-BTV-NS3 (group #1, 7 mice) or Ad5-BTV-VP2 (group #2, 5 mice) or Ad5-BTV-VP7 (group #3, 5 mice), Ad-DsRed (6 mice) or PBS (3 mice). IU for the adenoviral stock has been calculated followed manufacturer indications (Adeno-XTM Rapid Titer Kit, Clontech). Briefly, dilutions of the adenovirus stock were used to infect HEK 293 cells. 48 hours later, these cells were fixed and stained with the antibody specific for the adenovirus hexon protein. Signal was detected after a secondary antibody conjugated with horseradish peroxidase (HRP) and DAB substrate. The infected cells turned dark brown and they were counted under a 20X objective. The stock titer has been calculated as: (infected cells/field) x (fields/well)/ volume virus (ml) x (dilution factor). They were challenged intraperitoneally with 10^3^ PFUs of BTV-8 two weeks after the last immunization, and were bled before each immunization and virus challenge, and were monitored daily for disease signs characterized by ocular discharges and apathy starting at 48 h post-infection. Disease progression included hunched posture, decreased food intake and lethargy. At this point, considered as humane endpoints in our animal protocols, mice were euthanized to stop pain or distress. The euthanize method used was by inhalant anaesthesia (Isoflurane) and cervical dislocation

### Sheep vaccination

One year old female Colmenareña breed sheep from a certified provider were accommodated in the Animal Facility at CISA-INIA, randomly divided in: group # 1, five sheep (numbered 1 to 5) were vaccinated intramuscularly (im) with 10^9^ IU of Ad5-BTV-VP7; group # 2, five sheep (numbered 6 to 10) were vaccinated with 10^9^ IU of Ad5-BTV-VP7 and 10^9^ IU of Ad5-BTV-VP2; group # 3, three sheep (numbered 11 to 13) were vaccinated with 10^9^ IU of Ad5-DsRed; and group # 4, three sheep (numbered 14 to 16) were administered with PBS. The animals received one booster with the same dose after 21 days. Challenge was performed intravenously with 10^6^ PFUs of BTV-8. Sheep were bled before each immunization and before challenge. Animals were daily examined for clinical signs of infection and rectal temperature was recorded. For all the experimental period, a clinical score was assigned daily to each sheep based on a clinical reaction index (CRI) as described [38]. These included attitude and lameness, feed intake, appearance of conjunctiva, scleral blood vessels and cornea, redness and haemorrhagic, nasal and ocular discharge, salivation, respiratory rate and sounds, oedema of the tongue and body temperature. The humane endpoint contained in our animal protocol for sheep and approved by our Committees (see above) included rapid or progressive weight loss, debilitating diarrhoea, dehydration, hunched posture, excessive or prolonged hyperthermia (>42°C), bleeding from any orifice and, in general, any condition interfering with daily activities (e.g. eating or drinking, ambulation or elimination). During all the experimental time any of the animals showed these humane endpoints to consider euthanizing. At the end of the experiment sheep were euthanized by intravenous administration of T61 (4–6 ml/50Kg bw) following intramuscular xylazona (0.3 mg/Kg bw) to minimize suffering.

### RT-qPCR specific for BTV segment 5

Total RNA was extracted from whole blood with Trizol Reagent Solution (Invitrogen) following manufacturer indications. The real time RT-qPCR specific for BTV segment 5 was performed as described [39].

### Histopathology

Lung samples were fixed in 4% buffered formalin (pH 7.2). After fixation, samples were dehydrated through a graded series of alcohol to xylol and embedded in paraffin wax. Sections of 4 μm thick were cut and stained with haematoxylin and eosin (H & E) for histopathological analyses.

### Anti-VP7/BTV IgG ELISA

MaxiSorp plates (Nunc, USA) were coated with 150 ng/well of baculovirus-expressed VP7 protein (gently provided by Dr. Javier Ortego, CISA-INIA) in carbonate buffer pH 9.3, overnight at 4°C. To detect BTV-specific IgGs in animals vaccinated with Ad5-BTV-VP2, plates were coated with BEI-inactivated BTV-8 [28] and incubated overnight at 4°C. Plates were subsequently washed with PBS 0.05% Tween-20 (PBS-T) and blocked with PBS-T containing 5% non-fat milk for 2h. Serial dilutions of the corresponding sera were performed in PBS-T containing 2% non-fat milk, plated and incubated for 1h. Signal was detected using donkey anti-sheep IgG-HRP (*ABD Serotec*) or goat anti-mouse IgG-HRP (*Biorad*) and developed with 3, 3’, 5, 5’- Tetramethyl-benzidine (TMB, *Sigma*). Signal development was stopped by the addition of 3M H_2_SO_4_ and absorbance read at 450nm in a Fluostar luminometer. Antibody titers were expressed as the reciprocal of the highest dilution giving an OD_A450_ of twice the value obtained with pre-immune serum for each animal.

### Virus neutralization test

Serum samples were tested for the presence of neutralizing antibodies as described elsewhere [40]. Briefly, serial dilutions of serum samples (1:2 to 1:256) were incubated with 100 PFUs of BTV-8 at 37°C for 1h in 96-well plates. 2x10^4^ Vero cells were subsequently added per well and incubated for 5 days. All dilutions were performed in duplicate and the neutralization titer is expressed as the reciprocal of the highest dilution of sera at which virus infection is blocked.

### Intracellular cytokine staining in mice

2x10^6^ splenocytes were stimulated with BEI-inactivated BTV-8 or the appropriate negative controls for 5h at 37°C 5% CO_2_ in 96-well plates, in presence of 1 μg/ml of Brefeldin-A (Sigma-Aldrich). Cells were harvested and stained with anti-mouse CD4-FITC and anti-mouse CD8-PerCP antibodies (BD Pharmingen). After washing and permeabilisation with PBS containing 0.1% saponin, cells were stained with anti-mouse IFN-γ-PE (BD pharmingen). The cells were acquired on a BD FACSCalibur flow cytometer (Becton Dickinson and Co., Franklin Lakes, New Jersey, USA) and analysed with FlowJo (Tree Star Inc., San Francisco, California, USA).

### PBMCs isolation from sheep and intracellular IFN-γ staining

Peripheral blood mononuclear cells (PBMCs) were obtained by standard gradient centrifugation method. Briefly, blood collected in EDTA (6mM final concentration) was diluted 1:1 in PBS + 0.03% (w/v) EDTA and overlaid over a Ficoll cushion (GE Health-care Europe GmbH). Blood was centrifuged at 800 x *g* for 30 min at room temperature without brake, and the PBMCs present at the interface were transferred to a fresh tube and washed with PBS+0.03% (w/v) EDTA. 2–3 x 10^5^ PBMCs/well were cultured in the presence of BEI-inactivated BTV-8 or negative controls for 5h at 37°C 5% CO_2_ in 96-well plates, in presence of 1 μg/ml of brefeldin-A (Sigma-Aldrich). Cells were harvested and stained with anti-ovine CD4-FITC and anti-ovine CD8-PE antibodies (Serotec). After permeabilisation, PBMCs were stained with anti-ovine IFN-γ-A647 antibody (Serotec). Cells were acquired on a BD FACSCalibur flow cytometer (Becton Dickinson and Co., Franklin Lakes, New Jersey, USA) and analyzed with FlowJo software (Tree Star Inc., San Francisco, CA, USA).

### Statistical analyses

The statistical analyses were done using unpaired two-tailed Student’s t-test. A tow-tailed non parametric Mann-Whitney U test was used to compare group of values from several animals. Data handling analyses was performed using Prism 6.0 (GraphPad Software Inc. San Diego, CA, USA).

## Results

### Generation of three recombinant adenoviruses expressing BTV-8 NS3, VP2 and VP7 proteins

**Fig 1:**
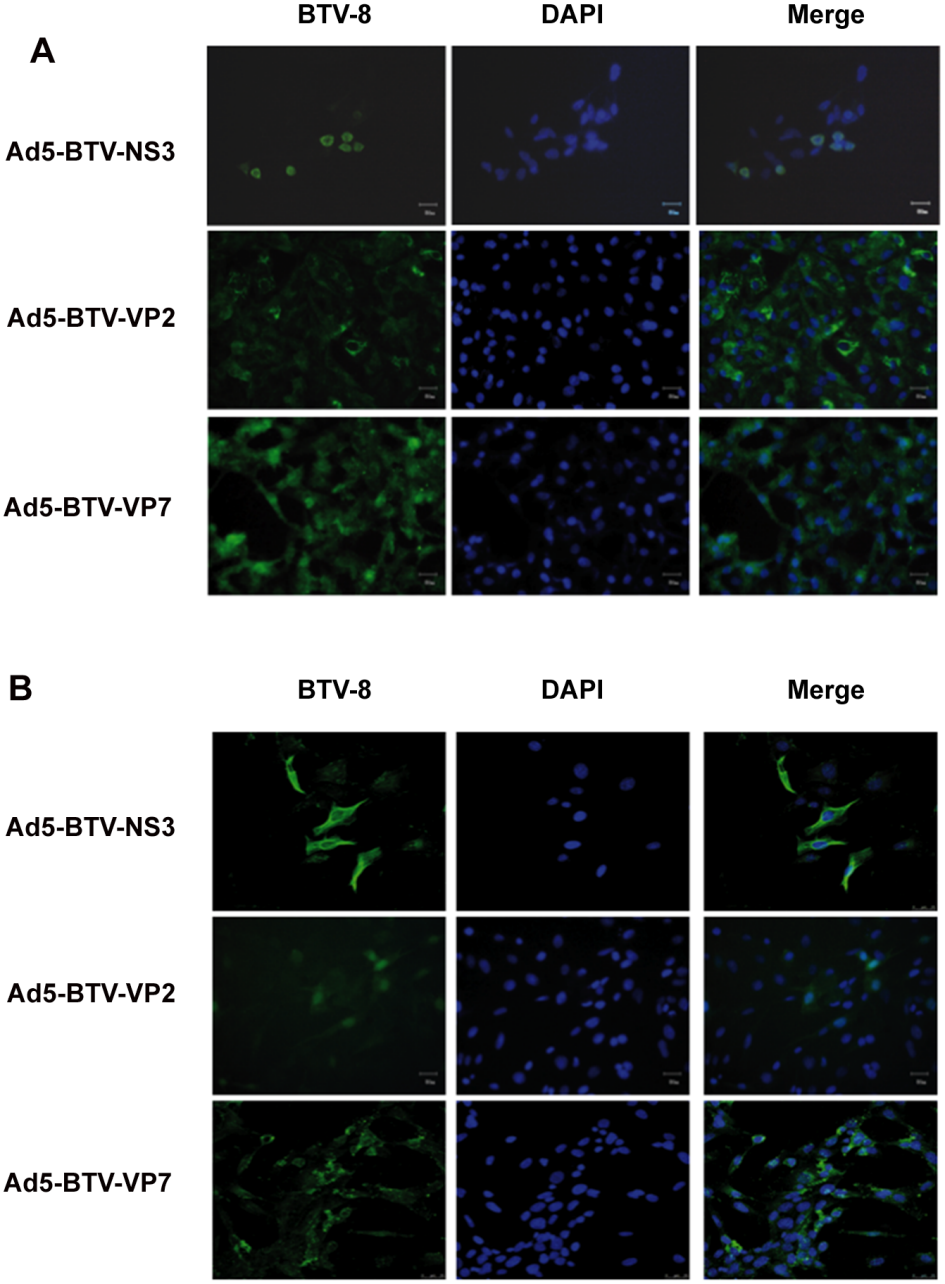
Expression of BTV-8- NS3, VP2 and VP7 proteins from recombinant adenoviruses in Vero and ST cells. Fluorescence microscopy was used to analyse the expression of NS3, VP2 or VP7 proteins after infection with recombinant adenoviruses Ad5-BTV-NS3, Ad5-BTV-VP2 or Ad5-BTV-VP7. (A) Vero cells infected with Ad5-BTV-NS3, Ad5-BTV-VP2 or Ad5-BTV-VP7. (B) ST cells infected with Ad5-BTV-NS3, Ad5-BTV-VP2 or Ad5-BTV-VP7. The three panels shown from left to right are antibody staining against BTV-8 (green), DAPI staining (blue), and merge. The micrographs are representative of at least three independent experiments. Magnification 20X; bar graphs correspond to 50 nm.

**Fig 2:**
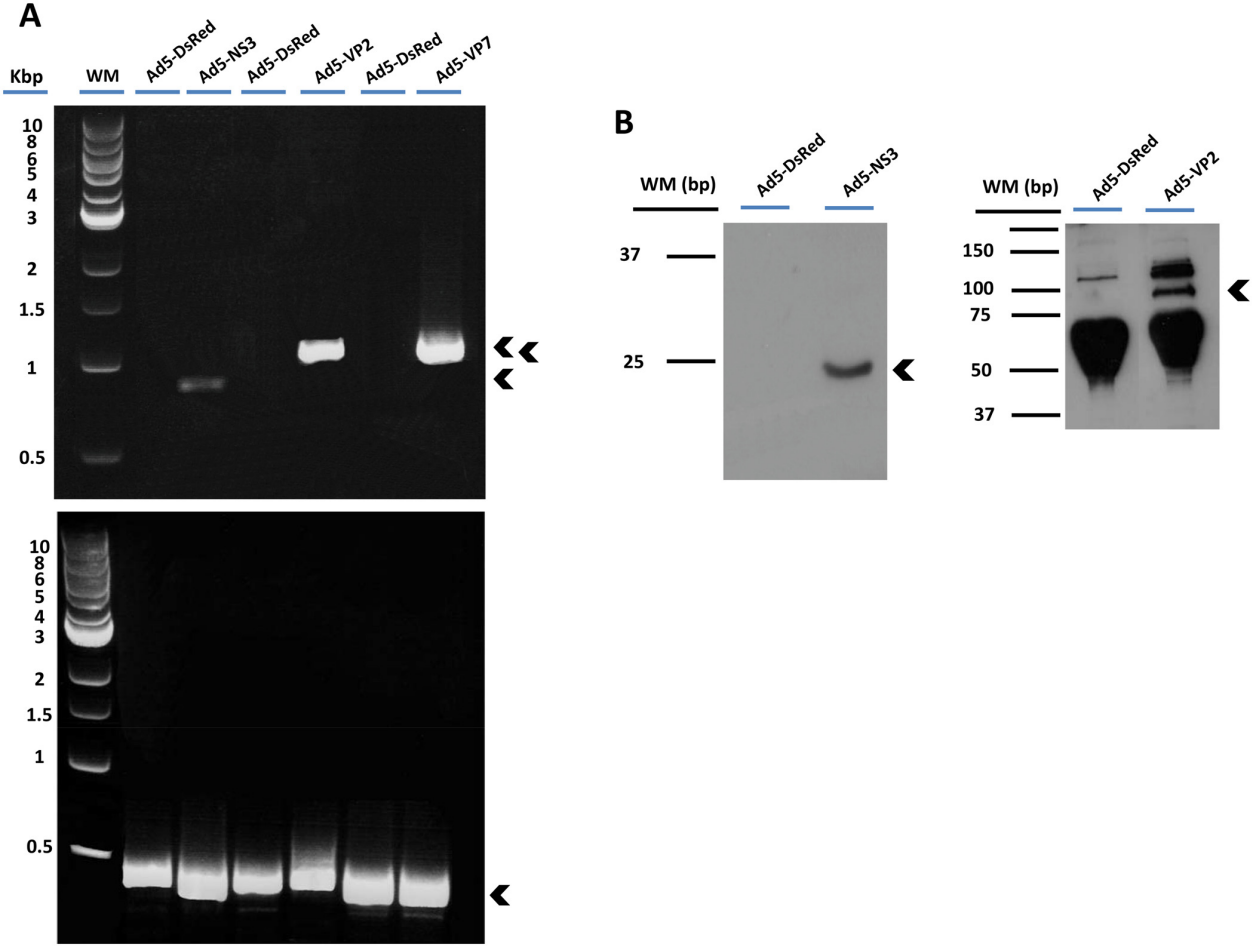
Expression of BTV-8 -NS3, -VP2 and -VP7 proteins from recombinant adenoviruses. (A) RT-PCR amplification from total RNA obtained from Ad5-BTV-NS3, Ad5-BTV-VP2, Ad5-BTV-VP7 and Ad5-DsRed infected HEK293A cells. Total RNA was assayed with a reverse transcription-PCR to detect NS3 (685 bp), VP2 (1042 pb) or VP7 (1084 bp) gene expression. Input RNA samples used are indicated above each lane. The bands corresponding to the proteins detected are indicated on the right (arrows). Primers pairs used are provided upon request. Molecular size markers (Kbp) are at right. As control, the expression of the cellular β-actin gene was determined by amplifying a 426 bp fragment of the corresponding gene by using actin-specific primers. (B) After adenovirus infection of HEK293A cells, expression of BTV-8 -NS3 (24 kDa) and -VP2 (112 kDa) proteins were analysed by Western blot using sera from BTV-8 infected mice or sheep. Input protein samples used are indicated above each lane. The bands corresponding to the proteins detected are indicated on the right (arrows). Molecular size markers (kDa) are at right.

Recombinant adenoviruses encoding NS3 (Ad5-BTV-NS3), VP7 (Ad5-BTV-VP7) and VP2 (Ad5-BTV-VP2) from BTV-8 were generated. To determine the expression of BTV proteins by recombinant adenoviruses, Vero and ST cells were infected and immunostained using serum from BTV infected sheep. Specific labelling corresponding to NS3, VP2 and VP7 proteins was detected in Ad5-BTV-NS3, Ad5-BTV-VP2, Ad5-BTV-VP7 infected cells, respectively (Fig 1A and 1B). As expected, no signal was observed in either Ad5-DsRed (parental control recombinant adenovirus) infected or uninfected cells using either antibody (S1 Fig). To confirm that infected HEK293A cells express the BTV-8 -NS3, -VP2 and -VP7 genes, we analysed the presence of the corresponding transcripts in these cells by RT-PCR, which were readily detected (Fig 2A). To detect the expression of BTV-8 -NS3, -VP2 and -VP7 proteins, western blot analysis using polyclonal sera from BTV-8 infected mice was performed. Specific labelling corresponding to BTV-8 -NS3 (26 KDa) and -VP2 (111 KDa) proteins was detected in Ad5-NS3 and Ad5-VP2 infected cells, respectively (Fig 2B). As expected, no signal was observed in Ad5-DsRed infected cells. The VP7 protein has a 37 KDa expected size. In this case and with our antisera is not possible to detect the VP7 expressed in the HEK293A infected with Ad5-VP7.

### Immunization with recombinant adenovirus induces protection against lethal challenge with BTV-8 in IFNAR^(-/-)^ mice

**Fig 3:**
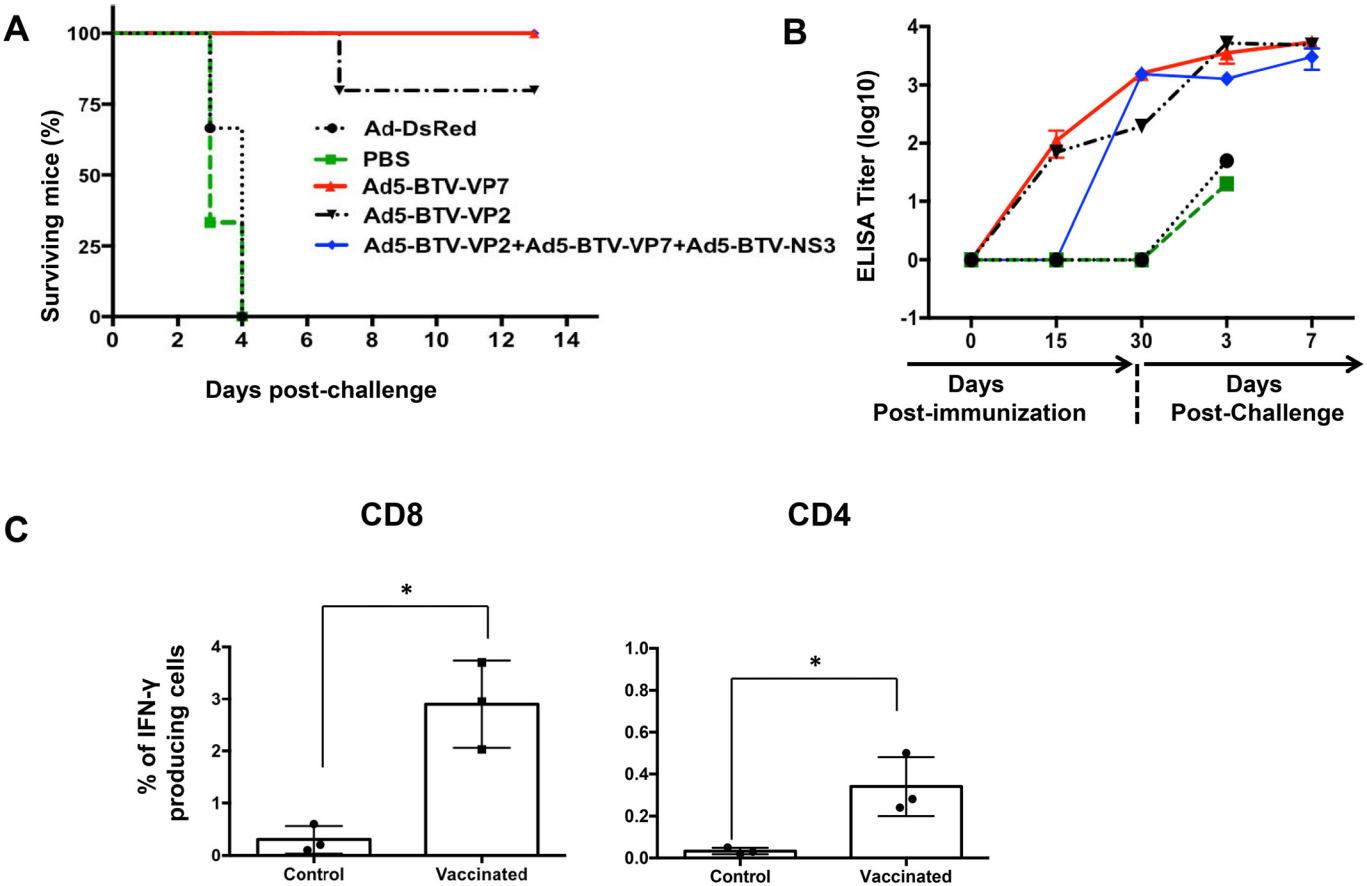
Immunogenicity of recombinant adenoviruses Ad5-BTV-NS3, Ad5-BTV-VP7 and Ad5-BTV-VP2 in IFNAR^(-/-)^ mice. Mice were immunized twice with recombinant adenoviruses (group#1: Ad5-BTV-NS3 + Ad5-BTV-VP7 + Ad5-BTV-VP2; group #2: Ad5-BTV-VP2; and group#3: Ad5-BTV-VP7). At D30 post-vaccination, 15 days after the booster injection, mice were challenged with 10^3^ PFUs of BTV-8. (A) Survival curves after challenge. (B) BTV-IgG titers in serum of mice immunized with recombinant adenovirus and after challenge (indicated with an arrow the day of challenge). (C) BTV-specific T cell responses in vaccinated sheep. Splenocytes from 3 mice from group #1 (vaccinated) and 3 mice from the control group inoculated with Ad5-DsRed were obtained 7 days after the booster vaccination and stimulated with BEI-inactivated BTV-8. Data show the percentage of CD8^+^ and CD4^+^ T cells producing IFN-γ detected by intracellular cytokine staining. * indicates statistically significant (p< 0.005, Wilcoxon test).

Adult IFNAR^(-/-)^ mice are a valid surrogate model to study the effectiveness of BTV vaccines [34]. Therefore, we used IFNAR^(-/-)^ mice as a model to evaluate the immunogenicity and capacity to confer protection against infection with BTV-8 of the Ad5-BTV-NS3, Ad5-BTV-VP2 and Ad5-BTV-VP7. Five groups of mice were inoculated with either Ad5-BTV-VP2 + Ad5-BTV-VP7 + Ad5-BTV-NS3 (group #1, 7 mice), Ad5-BTV-VP2 (group #2, 5 mice), Ad5-BTV-VP7 (group #3, 5 mice), Ad-DsRed (6 mice), or PBS (3 mice). Animals received a booster dose at day 15 post-priming and were challenged at day 15 post-booster with 10^3^ PFUs of BTV-8. Each mouse was bled before the booster vaccination and before challenge and at days 3, 5, 7, 10 and 12 post-challenge (pc) to determine levels of BTV-specific antibody titer and T-cell responses. All the control animals (mice receiving Ad-DsRed or PBS) died at day 4 post-challenge (pc) (Fig 3A), as expected [34]. In sharp contrast, none of the mice vaccinated died or developed any signs associated with BTV replication (Fig 3A) but one mouse of group #2 (vaccinated with Ad5-BTV-VP2) that died at D7 pc. BTV-specific IgG levels increased following immunization in vaccinated groups (Fig 3B) to get high levels before challenge. To evaluate the induction of BTV-specific T-cell responses as a result of vaccination, 3 mice of group #1 were chosen because they were inoculated with the 3 rAds, and 3 mice from the control group, inoculated with Ad-DsRed. These were sacrificed at 7 days after the booster injection and splenocytes were cultured in the presence of BEI-inactivated BTV-8. T-cell responses were measured using flow cytometry analyses for intracellular IFN-γ staining. As shown in Fig 3C, a significant percentage of CD8^+^ and CD4^+^ T cells from vaccinated mice were producing IFN-γ (p<0.005, Wilcoxon test). These data indicate that mice immunized with Ad5-BTV-VP2 + Ad5-BTV-VP7 + Ad5-BTV-NS3 (group #1) were able to elicit both humoral and cellular immune responses against BTV which was potent enough to confer protection.

### Sheep immunized with recombinant adenoviruses were protected against challenge with BTV-8

**Fig 4:**
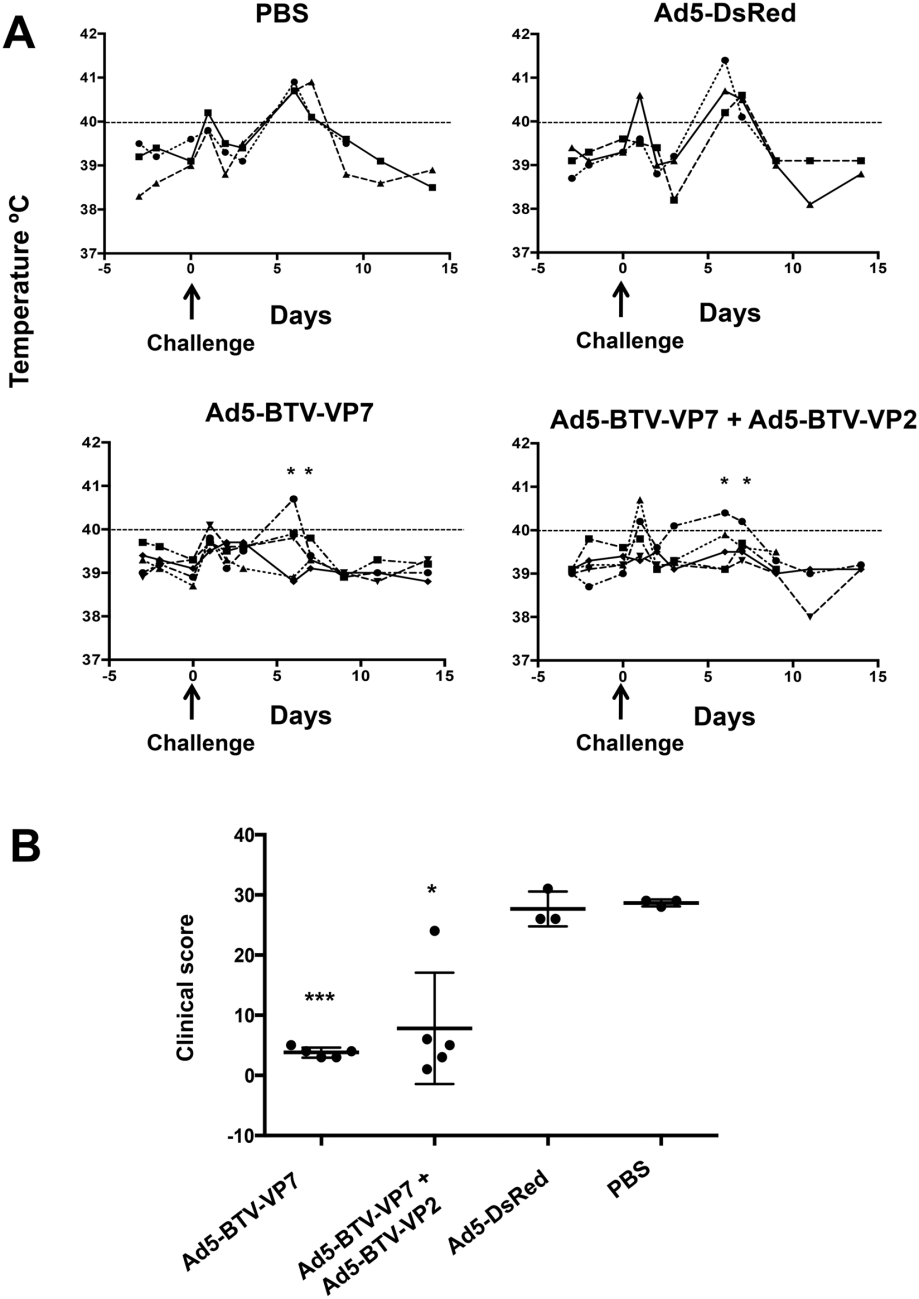
Rectal body temperature and clinical signs of vaccinated sheep with recombinant adenovirus. (A) The rectal temperature was recorded daily for a total period of 14 days. Temperature for individual animals in each group is shown. Fever was considered when temperature was above 40°C (indicated with dotted line). The asterisks indicate statistically significant at peak of fever (D6 and D7 pi) (p = 0.0009 for Ad5-BTV-VP7 and p = 0.0036 for Ad-BTV-VP7+Ad5-BTV-VP2) by unpaired t test with Welch’s correction. (B) Clinical signs recorded in vaccinated and control sheep after challenge with BTV-8. Sheep were scored daily using a clinical index score that takes into account general symptoms, fever, respiratory signs or death as described in Material and Methods. Total scores are given for individual animals as the accumulative values for each symptom collected through the observation period. The asterisks indicate statistically significant (* p = 0.0194; *** p< 0.0001) by Wilcoxon Test.

The efficacy of the new generated adenoviruses as a BTV vaccine in IFNAR^(-/-)^ mice encouraged us to investigate their efficiency in sheep. Ad5-BTV-VP2 and Ad5-BTV-VP7 were chosen based on their capacity to induce humoral and T cell responses as well as because the lack of NS3 directed antibodies by the Ad5-BTV-VP2 or Ad5-BTV-VP7 vaccine may enable differentiation of infected from vaccinated animals (DIVA principle). Four different vaccination groups were used. In group # 1, five sheep (numbered 1 to 5) were vaccinated with Ad5-BTV-VP7; in group # 2, five sheep (numbered 6 to 10) were vaccinated with Ad5-BTV-VP7 and Ad5-BTV-VP2; in group # 3, three sheep (numbered 11 to 13) were vaccinated with Ad5-DsRed; and in group # 4, three sheep (numbered 14 to 16) were administered PBS. All groups received a booster injection at day 15 post-immunization with the same dose and antigen composition. Finally, animals were challenged at 15 days post-booster with BTV-8. During the experiment, clinical signs and rectal temperature were recorded (Fig 4 and S2 Fig). Sheep in both control groups showed increased temperature of >40°C at D5pc (Fig 4A), declining to normal values by D9pc. Vaccinated sheep challenged with BTV-8 did not show high temperature but one animal in group #1 (vaccinated with Ad5-BTV-VP7) that however did not develop any additional clinical sign and one animal in group #2 (vaccinated with Ad5-BTV-VP7 + Ad5-BTV-VP2) that developed clinical signs. All animals in the control groups developed clinical signs associated to BTV infection (see Material and Methods), with an average clinical score of 28.7 ± 2.7 (Fig 4B). In stark contrast, the animals vaccinated with Ad5-BTV-VP7 developed very mild clinical signs of disease (average score of 3.8 ± 0.5) but significantly reduced when compared with control groups (p value < 0.0001, Student’s t-test). Four sheep of the group vaccinated with Ad5-BTV-VP7 + Ad5-BTV-VP2 showed also reduced clinical signs but one animal developed clinical signs similar to control groups (clinical score of 24) (average clinical score of the group 10 ± 8.3, significantly lower that the control groups, p value = 0.0194, Student’s t-test. In addition to the observed clinical signs recorded daily, 3 sheep per group (1 from each control group) were sacrificed at day D9pc to evaluate the lesions presented post-mortem. All control sheep showed gross changes such as haemorrhages, ulceration of the mucosal of the upper gastrointestinal tract; haemorrhages in lymph nodes and pulmonary artery and pulmonary oedema as main lesions. Vaccinated animals (group #1 and #2) did not show any of these lesions. Histological examination of the lungs showed hyperaemia, severe alveolar oedema with interstitial cellularity in control animals whereas normal lung histology is found in vaccinated sheep (Fig 4A). Comparison of the results of vaccinated sheep with control groups suggests that the new adenoviral BTV vaccine protects sheep against BTV-8 challenge.

**Fig 5:**
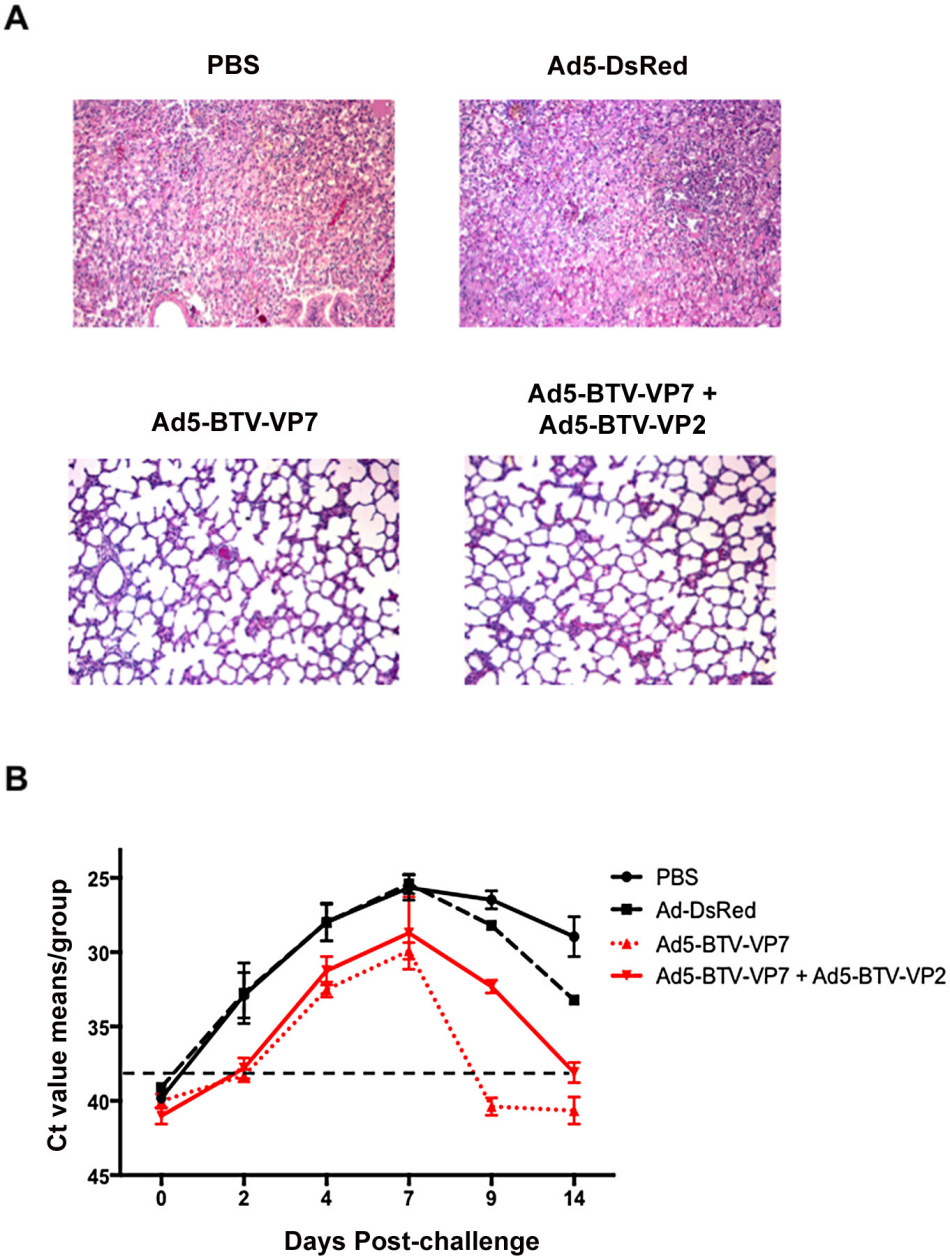
Histopathological lesions and viral load in vaccinated sheep. (A) Histopathological lesions in lungs sections stained with haematoxylin and eosin. Tissues were harvested at day 9 post-challenge with BTV-8. One representative animal is shown for each group. In control groups (PBS and Ad5-DsRed), pulmonary oedema with expansion of the interlobular septae and accumulation of protein rich fluid in airspaces was found. Magnification 100x. (B) Blood was collected from control (PBS and Ad5-DsRed) and vaccinated (Ad5-BTV-VP7 and Ad5-BTV-VP7 + Ad5-BTV-VP2) sheep at indicated times post-challenge with BTV-8. Total RNA was extracted from whole blood samples and RT-qPCR was performed as indicated in Material and Methods. Results are expressed as Ct. The cut off is indicated with a dotted line (Ct = 38 according to [39]).

To determine the level of protection, viremia was evaluated in all animals by RT-qPCR. All sheep developed viremia, but in the vaccinated animals (group # 1 and # 2) the onset of viremia was delayed, lasted for a shorter time (cleared by D9pc or D14pc), and the Ct value was higher than in the control groups at the peak of viral replication (Ct values from 25 in control groups to 29.3 in vaccinated animals at D7pc) (Fig 5B). In general, viral loads remain significantly lower (Ad5-BTV-VP7 + Ad5-BTV-VP2 vaccinated sheep p value < 0.0397 and Ad5-BTV-VP7 vaccinated sheep p value < 0.0004, Mann-Whitney U test) than those obtained in control animals. Taken all the data together, vaccination with Ad5-BTV-VP7 and Ad5-BTV-VP2 protected sheep of BTV-challenge although this vaccination did not induce sterile protection.

### Vaccination with Ad5-BTV-VP7 and Ad5-BTV-VP2 induces systemic BTV-specific antibodies but different levels of neutralizing antibodies

**Fig 6:**
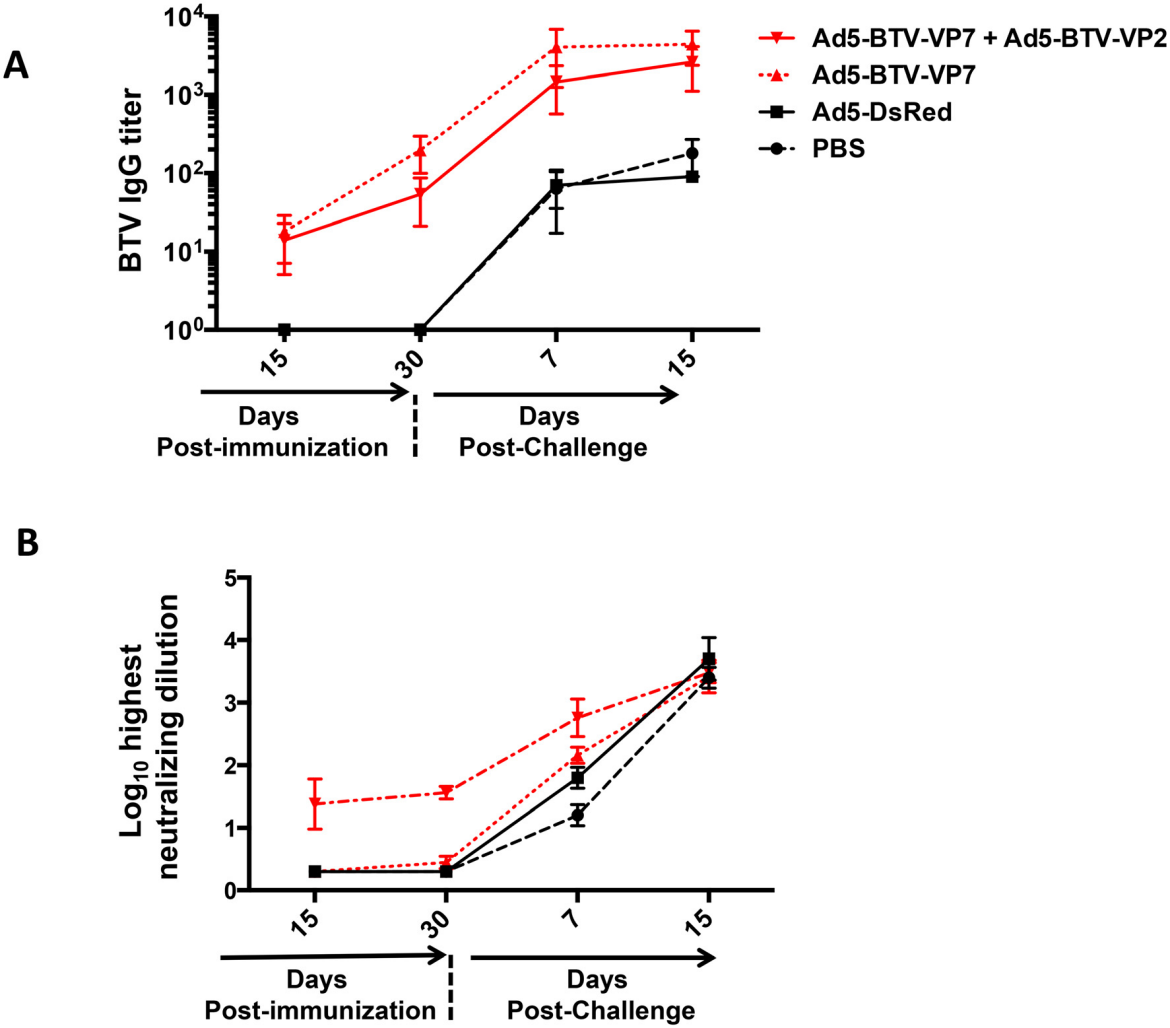
Serum IgG and neutralizing antibodies response in sheep following immunization with recombinant adenovirus. (A) Antibody responses elicited against VP7 protein assessed by ELISA. Data are expressed as the mean ± SD of antibody titers of individual values obtained for each animal. Titers are expressed as the reciprocal of the highest dilution of serum (log_10_) that gives an OD_A450_ of twice the value obtained with the pre-immune serum of the corresponding animal. (B) Neutralizing antibody response after vaccination and after challenge of the vaccinated and control sheep. The neutralizing antibodies titers are given in log_10_. The detection limit of the assay is 0.3.

BTV-specific antibodies were quantitated by ELISA in serum of sheep vaccinated with recombinant adenovirus. IgG levels increased from day 15 post-vaccination in those animals vaccinated with Ad5-BTV-VP7 or Ad5-BTV-VP7 + Ad5-BTV-VP2 (Fig 6A), to D7pc, while sheep treated with PBS or vaccinated with Ad5-DsRed remained negative before challenge. This data indicates that all recombinant antigens were immunogenic, inducing the production of BTV-8-specific antibodies.

Therefore, we carried out neutralization assays to determine the level of neutralization activity against BTV-8. BTV-specific neutralizing antibodies were detected by day 15 post-vaccination in sera from animals vaccinated with Ad5-BTV-VP7 + Ad5-BTV-VP2 (group #2) and displayed similar, slightly higher neutralization titers by D30 post-vaccination (average of 1.38 ± 0.4 at D15 and 1.56 ± 0.1 at D30). Interestingly, BTV-specific neutralizing antibodies were not detected in sera from sheep vaccinated with Ad5-BTV-VP7 neither at day 15 post-vaccination nor at day 30 post-vaccination (Fig 6B). Taken together, these data suggest that only anti-VP2 antibodies showed BTV-8 neutralization activity *in vitro*.

### Recombinant adenoviruses induce a VP7-specific T cell response

**Fig 7:**
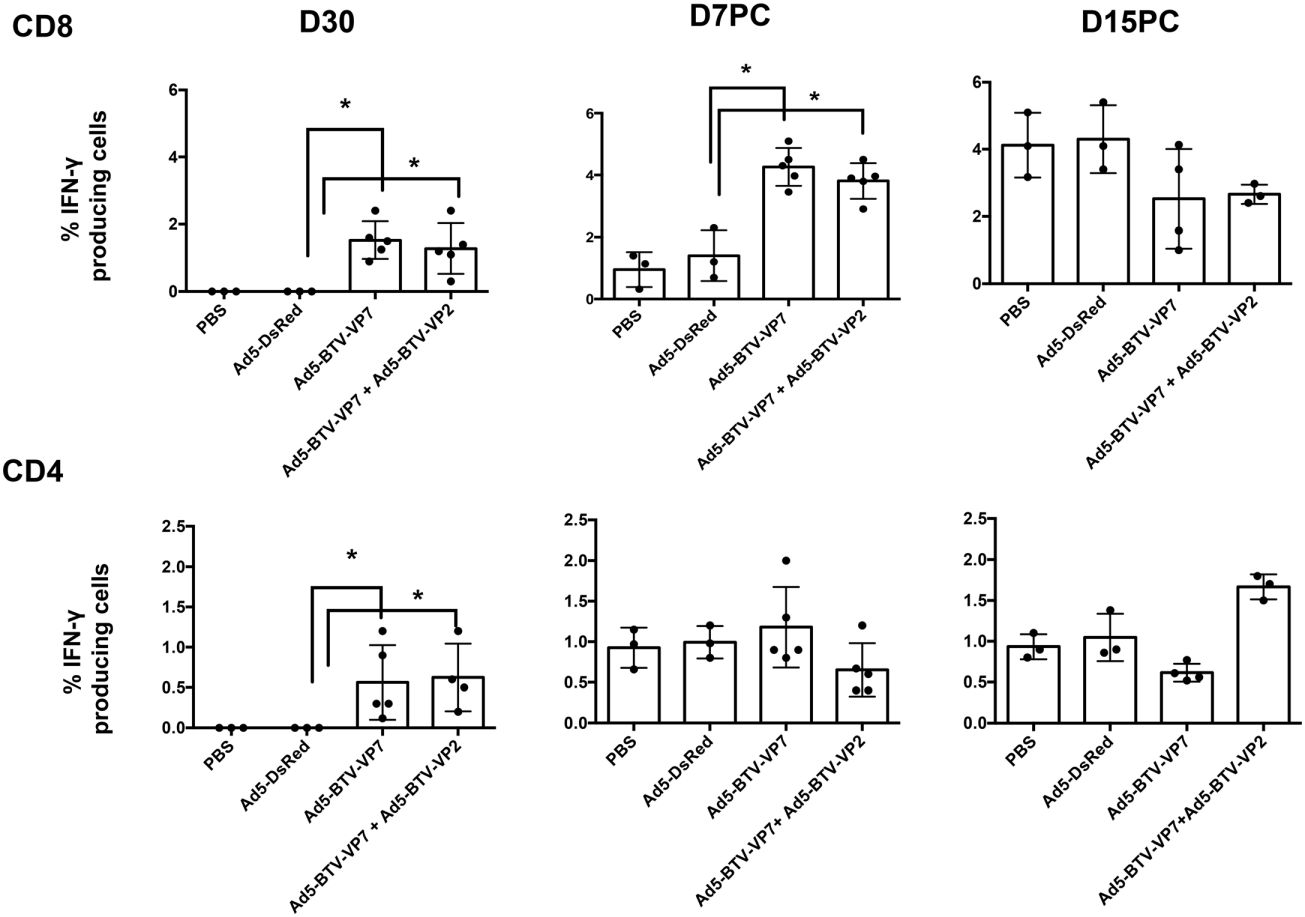
VP7-specific T cell responses in vaccinate sheep. PBMCs were purified from blood of vaccinated and control sheep at D30 post-vaccination (day of challenge), D7 pc and D15 pc, and stimulated with VP7 protein. Cells were stained for surface CD8 or CD4 and intracellular IFN-γ and analysed by flow cytometry. The percentage of CD8^+^ or CD4^+^ T cells producing IFN-γ after subtracting the background of the assay (response to RMPI medium) is indicated. Each dot corresponds to individual animals. Bars indicate the average of all animals. The asterisks indicate statistically significant with a p value of < 0.005 (Mann-Whitney U test).

To evaluate VP7-specific T cell responses, PBMCs from vaccinated and control sheep were taken at D30 post-vaccination (day of challenge), D7 pc and D15 pc, and the cellular immune responses against BTV was analysed by intracellular IFN-γ staining. In vaccinated sheep (groups #1 and # 2), a statistically significant (p< 0.05, Mann-Whitney U test) percentage of CD8^+^ T cells responded to BTV stimulation *in vitro* producing IFN-γ (Fig 7) at D30 post-vaccination. Interestingly, at D7pc the VP7-specific CD8^+^ T cell response in vaccinated groups was also significantly (p < 0.05, Mann-Whitney U test) higher than in control groups (average of 1.4 ± 0.6 in Ad5-DsRed treated sheep versus 4.2 ± 0.5 in group#1 and 3.8 ± 0.5 in group #2). By D15pc all groups showed similar levels of CD8^+^ T cells producing IFN-γ. Thus, this data suggest that adenovirus expressing VP7 vaccination can rapidly (by D30 post-vaccination) elicit an efficient (measured by their capacity to produce IFN-γ) *ex vivo* detectable CD8^+^ T cells responses.

CD4^+^ T cell activation has also been evaluated. Sheep vaccinated in group #1 and # 2 also exhibited significantly elevated VP7-specific CD4^+^ T cells responses in PBMCs by D30 (p < 0.05, Mann-Whitney U test). By D7 pc all groups showed similar VP7-specific CD4^+^ T cell responses (Fig 7), suggesting that although vaccination with recombinant adenovirus expressing VP7 induces CD4^+^ T cell responses, the kinetic of this induction after challenge is not influenced by vaccination.

## Discussion

Humoral and cellular immune effector mechanisms mediate immune response against BTV (reviewed in [41]). However, immunization with conventional inactivated vaccines induces mainly neutralizing antibodies responses, whereas cross-protection between different strains rely mainly on the induction of T-cell immunity. In the present study we report the generation of a novel vaccine approach based on human replication-defective recombinant adenoviruses expressing VP7 or VP2 BTV proteins able to induce a potent humoral and T-cell mediated responses against BTV. Animals vaccinated with Ad5-BTV-VP7 developed CD4^+^ and CD8^+^ T cell responses against BTV without inducing significant neutralizing antibody response although they were partially protected against BTV-8 challenge. Indeed, these animals showed similar levels of protection that those vaccinated with Ad5-BTV-VP7 + Ad5-BTV-VP2 which developed a strong neutralizing antibody response. These results suggest that T cells can contribute crucially to protection against BTV infection in situations where humoral responses alone may not be protective.

Recombinant adenoviral vectors are useful vaccine candidates for induction of vigorous T-cell immunity. Indeed, rAd5 is one of the most potent adenoviruses used as a vaccine vector, able to express high and persistent antigen levels that correlate with a robust protective CD8^+^ T cell immunity [42]. In addition, rAd5 is a suitable candidate for livestock due to the absence of pre-existing immunity against this human recombinant adenovirus. Our previous results using rAd5 to deliver H and F encoding sequences from peste des petits ruminants virus (PPRV) showed that vaccinated animals developed neutralizing antibodies as well as specific PPRV T-cell responses [35, 43]. Based on that, we have chosen rAd5 to deliver immunogenic BTV proteins that have been selected based on their capacity to induce specific neutralizing responses (VP2) [44] and cross-serotype T cell responses (VP7 and NS3) [16, 28]. Although NS3 has been considered as a low immunogenic BTV protein, the difficulties to get stable NS3 protein expression might underestimate the detection of NS3-specific responses [45]. In addition, while both outer capsid proteins VP2 and VP5 provide targets for neutralizing antibodies, some reports indicate that immunization with VP2 alone is sufficient to induce protective immune responses in vaccinated animals [26, 31, 46]. Only if the expression level of VP2 by the vector vaccine is low, the proportion of properly folded VP2 might be increased by the presence of VP5 due to their conformational interaction [47]. Since a good expression of VP2 is obtained with Ad5, the amounts of VP2 might be sufficient to induce neutralizing antibodies, and therefore we have not included VP5 in our vaccination approach. The present study indicates that the three rAd5 generated, Ad5-BTV-VP7, Ad5-BTV-VP2 and Ad5-BTV-NS3 express VP2, VP7 and NS3 respectively (Figs 1 and 2), indicating that rAd5 provide a useful platform to express BTV proteins for vaccination. An alternative approach would have been to express two or more BTV proteins in a poly-cistronic single adenoviral vector to avoid inoculation with several adenoviruses. This might represent an advantage for vaccination to avoid several shoots but since the potential of an adenovirus vector relies on the antigen expression level, in a poly-cistronic system the level of expression of the second gene depends of the expression of the first gene. Thus, we selected to vaccinate with individual adenoviruses expressing different genes.

IFNAR^(-/-)^ mice have been shown to be a good surrogate model to test BTV vaccines [34]. Therefore, we initially tested the protection efficiency of Ad5-BTV-VP7, Ad5-BTV-VP2 and Ad5-BTV-NS3 against a lethal challenge with BTV-8. Our results showed that vaccination with the three rAd expressing VP2, VP7 or NS3 was not critical for the induction of protection since vaccination with Ad5-BTV-VP2 or Ad5-BTV-VP7 alone was enough to protect mice. These results contrast with previous studies in which vaccination with MVAs (modified vaccinia virus Ankara) or DNAs expressing VP7 alone did not protect IFNAR^(-/-)^ mice against BTV-8 challenge [48], although animals showed a delay in disease onset. This might be explained by the use of rAd5 to express VP7. Thus, during vaccination with MVAs the presence of type I IFN enhances induction of CD8^+^ T cell responses [49], similar to protein subunits vaccination in which adjuvants are needed to induce a robust IFN induction to increase antigen uptake and enhance T cell immunity [50, 51]. By contrast, IFN-I signalling is not required for induction of CD8^+^ T cell immunity with rAd5 [42], suggesting that Ad5-BTV-VP7/NS3 induces a T cell response of greater magnitude than other recombinant vectors which might drive protection.

Based on the data obtained in mice, sheep were vaccinated with Ad5-BTV-VP2 alone or together with Ad5-BTV-VP7 but Ad5-BTV-NS3 was excluded. We have observed that the immune response elicited by these vaccines was not significantly better, or even seems to be less immunogenic in mice, when Ad5-BTV-NS3 was included. Therefore, Ad5-BTV-NS3 was not included in the vaccination trial and this provide the system with the advantage of enable differentiation of infected from vaccinated animals (DIVA principle) due to the lack of NS3 directed antibodies by the Ad5-BTV-VP7 or Ad5-BTV-VP2 vaccine. In both groups clinical signs and body temperature were significantly diminished compared with the control group but failed to inhibit viral replication entirely. Given the complex interaction of BTV, host and *Culicoides* vectors in the life cycle of infection, viremia should be kept to a minimum to avoid field transmission of the virus. Our vaccine, although failed to control viremia completely and this may represent a problem for transmission, the values of Ct obtained at the viremia peak in vaccinated animals were significantly lower (by 32-fold) than in non-vaccinated sheep, and the virus was cleared by D9pc in animals vaccinated with Ad5-BTV-VP7 and by D14 pc in those vaccinated with Ad5-BTV-VP7 + Ad5-BTV-VP2. This difference in viral clearance by the two vaccinated groups might be due to the quality of the T cell response induced by these vaccines. Although IFN-γ production is a marker of T cells functionality, more information about the T cell quality of such response might be obtained by measuring the ability of antigen specific cells to produce multiples cytokines. In this respect, we are currently investigating the cytokine profile of these T cells in vaccinated animals with the aim of understanding the immune response elicited by these vaccines that makes a difference in viral clearance. Importantly, vaccinated sheep did not show any gross lesion or histopathology associated with BTV. All these data together indicate that vaccination with Ad5-BTV-VP7 + Ad5-BTV-VP2 or with Ad5-BTV-VP7 induces non-sterile protection in sheep. This level of protection observed corresponds with diverse humoral and cellular responses. Both groups of vaccinated sheep developed BTV-specific antibody by D15 post-vaccination that is boosted by D30 post-vaccination. However, neutralizing antibodies were not detected after immunization with Ad5-BTV-VP7 although sheep were partially protected against BTV-8 challenge. Vaccination with recombinant capripox virus expressing VP7 also resulted in partial protection in the absence of neutralizing antibodies [27], suggesting that other mechanisms might mediate protection.

Despite the lack of neutralizing antibodies, Ad5-BTV-VP7 was able to prime CD8^+^ T cell responses against BTV-8, which expanded significantly as measured by CD8^+^ T-cell specific production of IFN-γ before and after BTV-8 challenge. This data suggests that an early T cell activation might correlate with an improved protective immune response and, even in a CD8^+^ T cell recall response by re-infections by BTV, the amplitude of the previous CD8^+^ T cell burst during vaccination may generate a higher number of memory T cells [52]. Further analyses of BTV-specific effector and memory CD8^+^ T phenotypes elicited by our vaccine formulations should be carried out to confirm this correlation. CD4^+^ T cell responses were also activated by Ad5-BTV-VP7, suggesting that CD4^+^ T-cell help might be acting in a primary stage of the CD8^+^ T cell response. Additional experiments are required to demonstrate whether CD8^+^ T cell maintenance after vaccination/infection may be reliant on signal from CD4^+^ T cells.

In summary, this study demonstrates that rAd5 can efficiently express BTV proteins and induce non-sterile protection against homologous challenge. Our findings have therefore important implications for vaccine design, as they suggest that inducing cellular responses during vaccination may maximize protective efficacy to BTV. It will interest to determine cross-protection between different BTV serotypes using this new human Ad5 approach as vaccine approach.

## Supporting Information

S1 Fig Immunofluorescence controls. Vero (A-D) cells and ST (I-L) cells infected with Ad5-BTV-NS3, Ad5-BTV-VP2, Ad5-BTV-VP7 and Ad5-DsRed in fluorescence microscopy detecting the DsRed protein expression. Vero (E) cells and ST (M) cells infected with Ad5-DsRed antibody staining (green), (F, N) DAPI staining (blue). Vero (G) cells and ST (O) cells uninfected antibody staining (green), (H, P) DAPI staining (blue). Magnification 20x; Bar graphs correspond to 50 nm.
(TIFF)

S2 Fig Rectal body temperature before challenge. The rectal temperature was recorded for a total period of 32 days before challenge. Temperature for individual animals in each group is shown. Fever was considered when temperature was above 40°C (indicated with dotted line).
(TIFF)
