# Supplementary figures and images for: Protective Efficacy in Sheep of Adenovirus-Vectored Vaccines against Bluetongue Virus Is Associated with Specific T Cell Responses
Source: PLoS One. 2015 Nov 30;10(11):e0143273. doi: 10.1371/journal.pone.0143273 (PMC4664254; doi:10.1371/journal.pone.0143273)

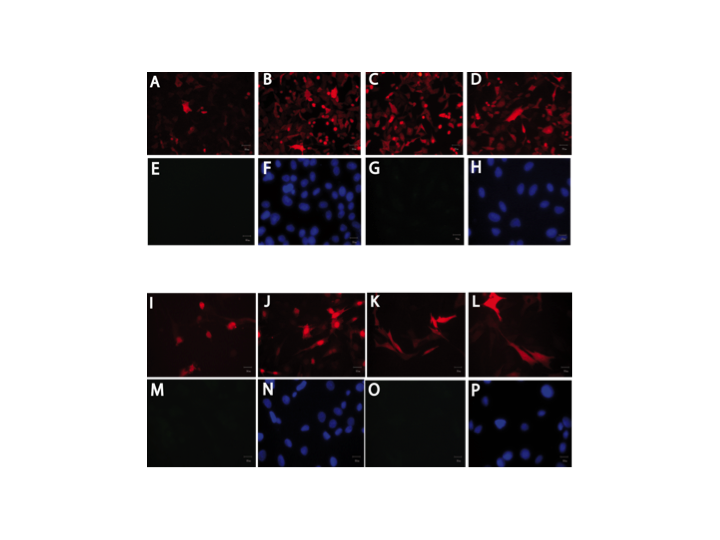

Supplement: S1 Fig — Vero (A-D) cells and ST (I-L) cells infected with Ad5-BTV-NS3, Ad5-BTV-VP2, Ad5-BTV-VP7 and Ad5-DsRed in fluorescence microscopy detecting the DsRed protein expression. Vero (E) cells and ST (M) cells infected with Ad5-DsRed antibody staining (green), (F, N) DAPI staining (blue). Vero (G) cells and ST (O) cells uninfected antibody staining (green), (H, P) DAPI staining (blue). Magnification 20x; Bar graphs correspond to 50 nm. (TIFF) [file pone.0143273.s001.tiff]

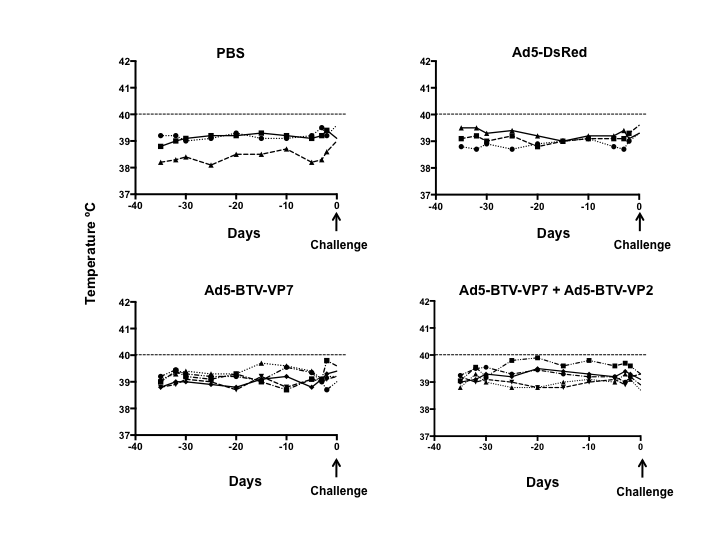

Supplement: S2 Fig — The rectal temperature was recorded for a total period of 32 days before challenge. Temperature for individual animals in each group is shown. Fever was considered when temperature was above 40°C (indicated with dotted line). (TIFF) [file pone.0143273.s002.tiff]
